# Resumption of traditional drive hunting of dolphins in the Solomon Islands in 2013

**DOI:** 10.1098/rsos.140524

**Published:** 2015-05-06

**Authors:** Marc Oremus, John Leqata, C. Scott Baker

**Affiliations:** 1 South Pacific Whale Research Consortium, 16 rue Henri Niautou, Nouméa 98800, New Caledonia; 2 Ministry of Fisheries and Marine Resources of the Solomon Islands Government, PO Box G2, Honiara, Solomon Islands; 3 Marine Mammal Institute and Department of Fisheries and Wildlife, Hatfield Marine Science Center, Oregon State University, 2030 Southeast Marine Science Drive, Newport, OR 97365, USA

**Keywords:** traditional hunting, small cetaceans, marine bushmeat, DNA barcoding

## Abstract

The ‘drive hunting’ of dolphins has a long history in the Solomon Islands, specifically at the island of Malaita. In 2010, the most active village, Fanalei, suspended hunting in exchange for financial compensation from an international non-governmental organization but resumed hunting again in early 2013. Here, we report on a visit to Fanalei in March 2013 to document the species and number of dolphins killed in the renewed hunting. Detailed records for the 2013 hunting, up to the time of our visit, included at least 1500 pantropical spotted dolphins (*Stenella* *attenuata*), 159 spinner dolphins (*Stenella longirostris*) and 15 ‘bottlenose’ dolphins, probably *Tursiops truncatus*. Molecular identification confirmed two of the species, pantropical spotted and spinner dolphins. A summary of all available records from 1976 to 2013 documented a minimum total of 15 454 dolphins killed by the Fanalei villagers alone. We also found the local price of a dolphin tooth had increased from about US$0.14 (SBD$1) in 2004 to about US$0.70 (SBD$5) in 2013. The large number of dolphins killed and the apparent incentive for future hunting offered by the increasing commercial value of teeth, highlight an urgent need to monitor hunts and assess the abundance and trends in local populations.

## 2. Introduction

The Solomon Islands, in the western South Pacific, are well known for the practice of dolphin hunting, where hunters use unique traditional techniques to drive schools from the offshore into shallow coastal waters [1–3]. Hunters operate in close coordination from 20 to 30 traditional canoes and, when dolphins are found, clap together rounded stones to create a percussive underwater sound [3]. The hunters manoeuvre the canoes into a ‘U’ shape around the dolphins while using the sound as an acoustic barrier to drive the dolphins towards shore where they are killed. Although a number of species have been hunted in the Solomon Islands [2,4], spinner dolphins *Stenella longirostris* and pantropical spotted dolphins *Stenella attenuata* have been the most common species targeted since the 1960s [2,4,5]. It is important to note that the controversial live-capture export trade of dolphins, initiated by the Solomon Islands in 2003, does not relate directly to traditional drive hunting. This export trade is focused on the capture of Indo-Pacific bottlenose dolphin (*Tursiops aduncus*), a species that is not targeted by hunters, presumably because it does not respond to traditional driving techniques [6].

The main objective of the drive hunt is to obtain teeth that are used as traditional currency, bride price, adornment and, more recently, for cash sale. However, the meat from the carcasses is also consumed, either within the hunting villages or after being sold locally on other islands and especially Honiara, Guadalcanal [7]. The practice of drive hunting was first documented during the early twentieth century [8], but it remains unclear when and where the hunt was initiated or introduced into the culture. Based on oral history, it most likely predates the arrival of the first missionary in the mid-nineteenth century [4]. However, it could potentially be much older.

Although there is a general perception that drive hunts for dolphins are conducted throughout the Solomon Islands, in fact this traditional practice is largely confined to a few villages on the island of Malaita and involves only Lau-speaking people. The dynamics of the hunt through history are not clear (e.g. which village went hunting and when) but previous reports indicate that it varied substantially over the years. Originally, the drive hunts did not occur annually [2]. Oral history from Fanalei village indicates that the dolphin hunt might have stopped around the middle of the nineteenth century, maybe in relation to the introduction of Christianity [4]. However, hunting was revived at Fanalei in 1948 by William Masura, the local vicar and then introduced anew at other Lau villages (Walande, Ata'a, Felasubua, Sulufou and Bita'ama) by Father Martin Fia in 1958 [3]. In their review, Reeves *et al.* [5] also reported that dolphin hunting stopped some time before World War II. Dolphin hunts used to be widely distributed in time, with probably some years between the hunts. However, in 1964, the scale of hunting increased markedly, resulting in catches of several thousand dolphins per year [2]. This expansion could be due to an increased market demand for dolphin teeth, resulting from a move towards a cash economy and the availability of Australian currency [5]. As of the mid-1960s, some of the villages had stopped the hunt while others continued, including Lau-people from North and South of Malaita [5]. According to Takekawa [4], Fanalei was the primary dolphin-hunting village and has continued hunting on a regular basis in more recent years.

In 2004, it was confirmed that the Bita'ama community (thought to be one of the primary hunting communities in the past) was not hunting for reasons that are unclear. During his discussions with elders from this community, Kahn [9] was told that the villagers would resume hunting soon. However, from our own discussions with Bita'ama elders, it seemed that hunting had not then resumed on a regular basis at the time of our earlier visit in July 2011. Note that the last hunts from Bita'ama community was apparently motivated by the recent development of the dolphin live-capture and export business in the country (Solomon Star News, 16 June 2009). This development aroused interest in the economic benefits of selling live dolphins (Solomon Star News, 5 June 2009).

During a previous visit to the Fanalei village in 2009, two of us (M.O. and J.L.) confirmed that this community was the last one to go hunting on a regular basis. However, in 2010, things took a new turn with the intervention of the non-governmental organization, the Earth Island Institute (EII) based in the USA. The EII offered financial support to develop alternative activities in exchange for stopping the hunt (Solomon Star News, 10 April 2010). A Memorandum of Understanding (MoU) was signed with Fanalei representatives, as well as with the former hunting communities of Bita'ama and Walande (M. Palmer, EII 2015, personal communication). These were also offered some financial support, although they had already stopped hunting for many years. However, in December 2012, the Solomon Star News reported that 134 ‘bottlenose dolphins’ were killed by the community of Ata'a,^1^ on the northeast of Malaita, using traditional methods. This community had no agreement with EII but is formally known to be a village with a tradition of drive hunting [4]. Soon afterwards, there was a breakdown of the agreement between the Fanalei villagers and EII (M. Palmer, EII 2015, personal communication) and on 22 January 2013, the Solomon Star News reported that the Fanalei community resumed hunting with a catch of 700 dolphins. According to later newspaper reports, this was soon followed by another catch of 300 dolphins (Solomon Star News, 25 January 2013).

The reported size of these catches raised concerns about the conservation status of these hunted populations, as well as concerns about animal welfare, given the presumed suffering experienced by these social species during the capture and killing [10]. Here, we report on our efforts to confirm the number and species identity of dolphins killed during recent hunting, by visiting Fanalei in March 2013.

## 3. Material and methods

Two of us (M.O. and J.L.) visited Fanalei on 22 March 2013 with the main objectives of confirming numbers of dolphins hunted during this season and identifying the species that were killed. To do so, we discussed local hunting with representatives, hunters and elders of the village at the community house. The meetings also provided us with an opportunity to discuss the community's future plans for hunting and conservation issues. J.L. acted as translator and recorded information on catches in his capacity as a Chief officer (Research) of the Ministry of Fisheries and Marine Resources. As well as discussions and reviews of catch records, we collected biological samples of the recent hunts to confirm traditional species names through species identification by molecular methods [11]. These samples were collected from four sources: (i) meat from recently hunted dolphins, found in the village's kitchens; (ii) skin from a few carcasses found at the dumping area; (iii) bone samples from the dumping area in the mangrove; and (iv) teeth from recent hunts, provided by villagers. However, given the variable state of decay and the limited time of our visit, no attempt was made to identify species using the morphology of the carcasses or skulls.

## 4. Results and discussion

### 4.1 Species identity

**Figure 1.**
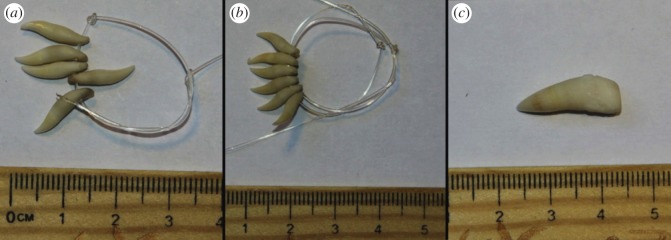
Teeth from the three species of dolphins hunted by the Fanalei in 2013: (*a*) ‘***unubulu***’, referring to spotted dolphin; (*b*) ‘***raa***’, referring to spinner dolphin and (*c*) young ‘***robo manole***’, probably referring to common bottlenose dolphin.

From our discussions at Fanalei, we were told that three species were included in the hunting, as of the time of our visit. These three species are locally known as ‘***unubulu***’, ‘***raa***’ and ‘***robo manole***’ (figure 1). The ‘***unubulu***’ and ‘***raa***’ clearly refer to the pantropical spotted dolphin and the spinner dolphin, respectively. Takekawa [4] suggested that ‘***robo manole***’ referred to the common dolphin (*Delphinus delphis*), but expressed some uncertainty about this identification. After questioning the hunters on the group size and morphological characteristics of the ‘***robo manole***’, it seemed more likely this name refers to the common bottlenose dolphin (*Tursiops truncatus*). We note that at the time of Takekawa's work [4], the taxonomic status of *Tursiops* in the Solomon Islands and elsewhere was still poorly known, probably contributing to some confusion with traditional names. Indeed, recent molecular identification based on biopsy samples collected at sea has confirmed the presence of two *Tursiops* species in the waters of the Solomon Islands: the larger common bottlenose, *T. truncatus*, found in deep offshore waters, and the smaller Indo-Pacific bottlenose, *T. aduncus*, found in shallow coastal waters [12]. Although Takekawa [4] considered that ‘***olo folosi walo***’ referred to *T. truncatus*, his description of small group sizes and inshore habitat use now suggest the species was in fact *T. aduncus*.

DNA was extracted from a total of 37 teeth, 11 meat samples and seven skin samples originating from dolphins taken in the drive hunt. We were able to amplify and sequence fragments of the mtDNA control region from eight of the teeth, nine of the meat samples and all seven skin samples. Overall, results confirm that drive-hunted dolphins are primarily spotted and spinner dolphins, as known from historical records. Of the eight teeth sequenced, five were spinner dolphins and three were of spotted dolphins. Unfortunately, no sequence could be obtained from one larger tooth thought to represent the common bottlenose dolphin, based on size and shape (figure 1). All of the samples of skin and meat were identified as spotted dolphins, consistent with reports of the hunt just before our visit to Fanalei.

### 4.2 Catch records for 2013

Catch records for the 2013 hunting season were provided to us by one of the dolphin hunters from Fanalei, Albert Balei, who kept detailed notes of the dates, species and number of dolphins caught during each hunt. These catches (*n*=11 hunting events) are summarized in table 1. It shows that the largest catch was the ‘***unubulu***’, or pantropical spotted dolphin, with over 1500 individuals taken. The second largest catch was the ***‘raa***’ or spinner dolphin, but in much smaller numbers with a total of 159 dolphins killed. Finally, a group of 15 ‘***robo manole***’, presumed to be common bottlenose dolphins, were caught. The average number of dolphins taken per event was 154+. It appears that there is a substantial difference between catches of pantropical spotted dolphins (mean of 218+ individuals per event) and spinner dolphins (53 individuals per event). The last hunt known to us was the day after our visit (23 March 2013) as reported to one of us (J.L.) through a Fanalei villager that visited Honiara soon after. Because the hunting season would usually continue through April [4], there could have been additional hunts of which we are not aware.

**Table 1.** Summary of dolphin catches by the Fanalei community from the beginning of the 2013 season until 23 March 2013, as reported by one of the dolphin hunters (A. Balei 2013, personal communication).

event	date	inferred species	traditional name	number caught
1	21 Jan 2013	*S. attenuata*	***unubulu***	700+
2	24 Jan 2013	*S. attenuata*	***unubulu***	60+
3	5 Feb 2013	*S. attenuata*	***unubulu***	126+
4	6 Feb 2013	*S. attenuata*	***unubulu***	300
5	9 Feb 2013	*T. truncatus?*	***robo manole***	15
6	11 Feb 2013	*S. longirostris*	***raa***	56
7	20 Feb 2013	*S. longirostris*	***raa***	33
8	20 Feb 2013	*S. attenuata*	***unubulu***	70
9	6 Mar 2013	*S. longirostris*	***raa***	70
10	20 Mar 2013	*S. attenuata*	***unubulu***	54
11	23 Mar 2013	*S. attenuata*	***unubulu***	214
			total	1698+

### 4.3 Total catch records, 1976–2013

In addition to the 2013 records, the Fanalei hunter, Albert Balei, also provided records of catches for the years 2000 to early 2003 season in Fanalei (table 2). Only pantropical spotted dolphins and spinner dolphins (‘***unubulu***’ and ‘***raa***’) were reported during this period, confirming the predominance of these species in traditional drive hunts. The number of successful hunts per year was: 10 in 2000, five in 2001 and 11 in 2002 (data were incomplete for 2003). The difference between the number of pantropical spotted dolphins and spinner dolphins caught was not as marked as in 2013 (728 versus 628, respectively, between 2000 and early 2003). On the other hand, the tendency for larger groups of pantropical spotted dolphins to be caught was confirmed, with a mean of 94 individuals hunt^−1^ for pantropical spotted dolphin compared with 42 individuals hunt^−1^ for spinner dolphin. However, we could not confirm whether entire schools were captured for any of the individual hunts.

**Table 2.** Summary of dolphin catches by the Fanalei community between 2000 and early 2003, as reported by one of the dolphin hunters (A. Balei 2013, personal communication).

year	event	date	species	numbers caught
2000	1	Sep 2000	*S. longirostris*^a^	15
	2	23 Jan 2000	*S. longirostris*	42
	3	15 Feb 2000	*S. longirostris*	15
	4	25 Feb 2000	*S. attenuata*	40
	5	2 Mar 2000	*S. longirostris*	55
	6	8 Mar 2000	*S. attenuata*	45
	7	27 Mar 2000	*S. longirostris*	44
	8	5 Apr 2000	*S. attenuata*	36
	9	6 Apr 2000	*S. attenuata*	274
	10	2 Dec 2000	*S. longirostris*	11
2001	1	31 Jan 2001	*S. longirostris*	19
	2	20 Feb 2001	*S. longirostris*	27
	3	17 Mar 2001	*S. longirostris*	54
	4	22 Mar 2001	*S. attenuata*	15
	5	24 Mar 2001	*S. longirostris*	16
2002	1	9 Jan 2002	*S. longirostris*	9
	2	4 Feb 2002	*S. longirostris*	96
	3	23 Feb 2002	*S. longirostris*	64
	4	6 Mar 2002	*S. attenuata*	18
	5	8 Mar 2002	*S. longirostris*	128
	6	18 Mar 2002	*S. attenuata*	50
	7	25 Mar 2002	*S. longirostris*	13
	8	30 Mar 2002	*S. longirostris*	33
	9	8 Apr 2002	*S. attenuata*	72
	10	9 Apr 2002	*S. attenuata*	40
	11	17 Apr 2002	*S. attenuata*	125
2003	1	14 Apr 2003	*S. attenuata*	400
			total	1756+

^a^Entered in the lagoon without driving.

Unfortunately, we were unable to obtain records for the 2003 to 2010 period, i.e. the period before the hunt temporally stopped. At that time, Albert Balei was away from the village and no one else seems to have kept accurate track of the catch records and hunt effort. However, Kahn [9] reported some overall annual catches for Fanalei for the period 1999–2004 (not 2001), which he collected during a visit to the community. For the year 2000, Kahn [9] reported a larger catch than the information given to us (800 versus 577), while for 2002, we were given fairly similar numbers (700 versus 648). The reason for the discrepancy in the numbers for the year 2000 is unknown. However, the records provided to us by Albert Balei seem to be otherwise accurate, including days not going to sea and days going out with no catch throughout the hunt season (electronic supplementary material, figure S1; and table S1).

**Figure 2.**
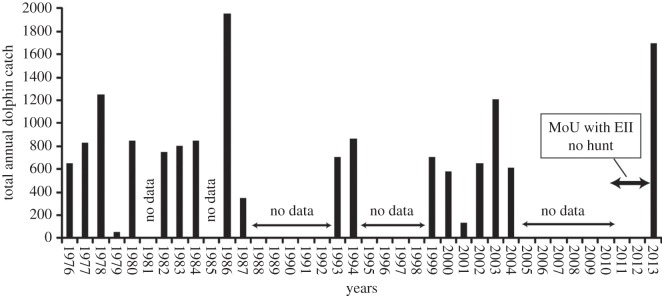
Temporal changes in the annual catches of dolphin by the Fanalei community between 1976 and 2013 (table 3). Years 1976 to 1994 from [4] and reference therein; years 1999, 2003 and 2004 from [9]; ears 2000, 2001, 2002 and 2013 from this study.

To the record from 2000 to 2003 and those from 2013, we added the historical records from Takekawa [3], covering the annual total catches at Fanalei for the period 1976–1994 (figure 2). These annual totals are based on D. Takekawa's 1993 [3] personal observations as well as on records he extracted from [13] and records reported to him by J. Filei, a community member from Fanalei. Takekawa [3] calculated an average of 840 dolphins taken per year during this period (maximum close to 2000 in 1986; minimum less than 50 in 1979). The average number of individuals caught per hunt was 115.5 (no details on the species) and the average number of successful hunts per year was 7.3. These numbers are roughly consistent with the new figures provided here. For instance, during the period 1999–2013, the mean annual catch was 793 dolphins, all species included.

Across the total period (1976–2013), a minimum of 15 454 dolphins were killed by the Fanalei villagers with a mean annual catch of 813 dolphins, s.d.=464 (table 3). However, this is clearly an underestimate of the number of dolphins hunted in the Solomon Islands, as we lack data for 16 hunting years across this period and only consider the community of Fanalei. If one considers that a usual annual catch would be between 600 and 1000 dolphins (i.e. ±200 around the average catch), it appears that success rate is fairly stable across years. The community had a lower success rate (less than 600 dolphins) for 3 years (1979, 1987 and 2001) while they had a high success rate (more than 1000 dolphins) for 4 years (1978, 1986, 2004 and 2013). There is no clear trend in success rate across years. We note that the two successful years for which data were available during the next season (e.g. 1978 and 1986) were followed by a low catch rate the following year (1979 and 1987, respectively). This tendency could indicate local depletion of dolphin populations following intensive hunting. We note evidence that at least one local population of melon-head whales, *Peponocephala electra*, was depleted by hunting in the Solomon Islands [4] and a similar case for local depletion of this species by hunting around the Island of Hawai'i in the nineteenth century [14]. Alternatively, the tendency for low years to follow high could be explained by a reduced need for dolphin teeth in the community, i.e. a reduced effort following successful hunting years. Unfortunately, the absence of species information for most years precludes any analyses of population-specific trend.

**Table 3.** Summary of annual catches of dolphins (all species) by the Fanalei community for the period 1976–2013, as known from different sources.

period	total annual catch	source
1976	650	Meltzoff [13] in Takekawa [4]
1977	825	Meltzoff [13] in Takekawa [4]
1978	1250	Meltzoff [13] in Takekawa [4]
1979	50	Meltzoff [13] in Takekawa [4]
1980	850	Meltzoff [13] in Takekawa [4]
1981	unknown	
1982	750	Takekawa [4]
1983	800	Takekawa [4]
1984	850	Takekawa [4]
1985	unknown	
1986	1950	Takekawa [4]
1987	350	Takekawa [4]
1988–1992	unknown	
1993	700	Takekawa [3]
1994	865	Takekawa [3]
1995–1998	unknown	
1999	700	Kahn [9]
2000	577	this study
2001	141	this study
2002	648	this study
2003	600	Kahn [9]
2004	1200	Kahn [9]
2005–2010	unknown	
2011–2012	no hunt	
2013	1698	this study
total	15 454	

### 4.4 Increasing market value of teeth

It is important to note that dolphin teeth are used by many villages in Malaita and not only by the hunting communities. Teeth (and meat) are also sent to other islands such as Guadalcanal and the Florida Islands. Indeed, it seems there is a high demand for this commodity and stopping the hunt had consequences that went beyond the village of Fanalei. Although adjusting for inflation of the Solomon Islands' currency is challenging, it appears that the price for dolphin teeth has increased beyond this expectation in recent years. Dawbin [2] reported that each tooth cost AUD$0.05 in 1964 (about US$0.06 or £0.02^2^), while Takekawa [4] reported the cost was set, in Solomon Islands Dollars, at SBD$0.50 per tooth in 1994 (about US$0.16 or £0.10). We could not find data on the Solomon Islands inflation rate before 1980 but the price increase between these two reports seems compatible with inflation since 1980.^3^ By 2004, Kahn [9] reported that the price had increased to SBD$1.00 per tooth (about US$0.14 or £0.07). According to inflation rates between 1994 and 2004 (about 238%), the price difference also seems in agreement with consumer price increase. The price for ‘***unubulu***’ and ‘***raa***’ teeth had remained little changed at the time of our first visit to Fanalei in 2009. However, by the time of our second visit in 2013, the price for ‘***unubulu***’ or ‘***raa***’ teeth had increased to SBD$5.00 per tooth (about US$0.68 or £0.45). The price of teeth from any ‘***robo***’ species (larger teeth) could even be higher but we were not given accurate figures. Based on a price of SBD$0.5 per tooth in 1994 [4] and annual inflation rates since that time, the price in 2013 should have been only about SBD$2.5 per tooth. The reason(s) for the unexpectedly higher prices in 2013 is unclear but it is possible that absence of hunts over the last 2 years created a higher demand for dolphin teeth.

The increase in the price of teeth could have been a factor in the decision by the village of Ata'a to resume hunting in December 2012. It was apparently the only hunt from this community this season and the first reported hunt in a very long time. From discussion with Fanalei elders about the hunt of 134 dolphins by Ata'a villagers in December 2012, we were told that the species caught was the pantropical spotted dolphin and not ‘bottlenose dolphins’, as reported in the local newspaper (Solomon Star News, 18 December 2012). There is indeed little reason to believe these were bottlenose dolphins (*Tursiops* sp.) as they are rarely taken and never with a school size this large. However, we were not able to collect any biological samples to confirm the species identity.

## 5. Conclusion and recommendations

It was our impression that the people of Fanalei were puzzled by the attention they attracted in resuming the recent dolphin hunt. To them, it seemed that the agreement with EII represented only a rather brief lapse in a long history of hunting. They explained that stopping the hunt had brought much tension in the village and that resuming it brought back peace among community members. Therefore, they made it clear that they intended to continue the hunt. However, it was also our impression that the hunters were aware of, and willing to discuss, the conservation implications of over exploitation. They also expressed concern about dolphin ‘by-catch’ by purse seiners in the Solomon Islands, seeing this as a threat to their local resource. They were not very receptive to the idea of introducing a quota or catch limit for the drive hunt, as they are concerned that would be too restrictive. On the other hand, they could see the value of collecting scientific data that might help increase the probability that the drive hunting could continue in future generations.

The International Union for the Conservation of Nature does not consider the primary species hunted in the Solomon Islands to be vulnerable or endangered: the pantropical spotted and common bottlenose dolphins are listed as ‘Least Concern’, the spinner dolphin is listed as ‘Data Deficient’. However, these species-wide threat classifications do not necessarily reflect the status or threats to local populations. The potential vulnerability of local populations in the Solomon Islands to over exploitation is suggested by recent evidence that several species, including the primary species targeted in drive hunts, tend to form small insular and genetically isolated populations around islands elsewhere in the Pacific [14–17]. Given this potential and our observation that the recent resumption of drive hunting showed no signs of abating, there is an urgent need to improve the monitoring of these catches, with the eventual objective of implementing a management procedure. First, there is a need to collect systematic records of all future hunts and, if possible, provide some verification through independent observers or photographic documentation. Second, samples from each hunt should be collected and archived, with the intent of confirming species identification and tracking changes in diversity and population identity over time, via genetic monitoring [18]. Finally, surveys of local waters are also needed to estimate the abundance of dolphins around Malaita. These recommendations are consistent with the Whale and Dolphin Action Plan developed by the Secretariat for the Pacific Regional Environment Programme [19], the inter-governmental agency responsible for providing management advice on cetaceans in the region.

In making these recommendations, we recognize that improved management of the hunt does not address the animal welfare concerns associated with drive hunting, although it might reduce the magnitude of the catches. Further reductions in catches might be achieved by providing an alternate, non-lethal value through dolphin-watching programmes or other ecotourism opportunities. Such programmes could take advantage of the local knowledge and skills available in the communities as a result of drive hunting, providing a more sustainable future for both the dolphins and the cultural traditions of the hunters.

## Supplementary Material

Figure S1: Example of catch records from notebook kept by local Fanalei hunter for the period 2000 to early 2003.

## Supplementary Material

Table S1: Catch records from notebook kept by local Fanalei hunter, Albert Balei, for the period 2000 to early 2003. Scientific names of dolphin species follow translations of local names as discussed in the text.

## Footnotes

1 There is no independent confirmation of species identity for this hunt.

2 All US$ and £ are based on historical exchange rates obtained from www.fxtop.com.

3 Adjustments for inflation were obtained from www.quandl.com.

## Data accessibility

The sequences used for identification of species from teeth and meat have been submitted to GenBank as KP756626 to KP756648.
