# Supplementary material for: Resumption of traditional drive hunting of dolphins in the Solomon Islands in 2013
Source: R Soc Open Sci. 2015 May 6;2(5):140524. doi: 10.1098/rsos.140524 (PMC4453245; doi:10.1098/rsos.140524)

Oremus M, Leqata J, Baker CS. 2015. ‘Resumption of traditional drive hunting of dolphins in the Solomon Islands in 2013’, *Proceedings of the Royal Society Open.*

Supplemental Material, Figure S1: Example of catch records from notebook kept by local Fanalei hunter, Albert Balei, for the period 2000 to early 2003. The symbol (x) indicates that no boat went out; (•) indicates that boat went at sea but found no dolphins; (∆) indicates that dolphins were sighted but there was no catch. Local species name and total number of dolphins are indicated when caught.


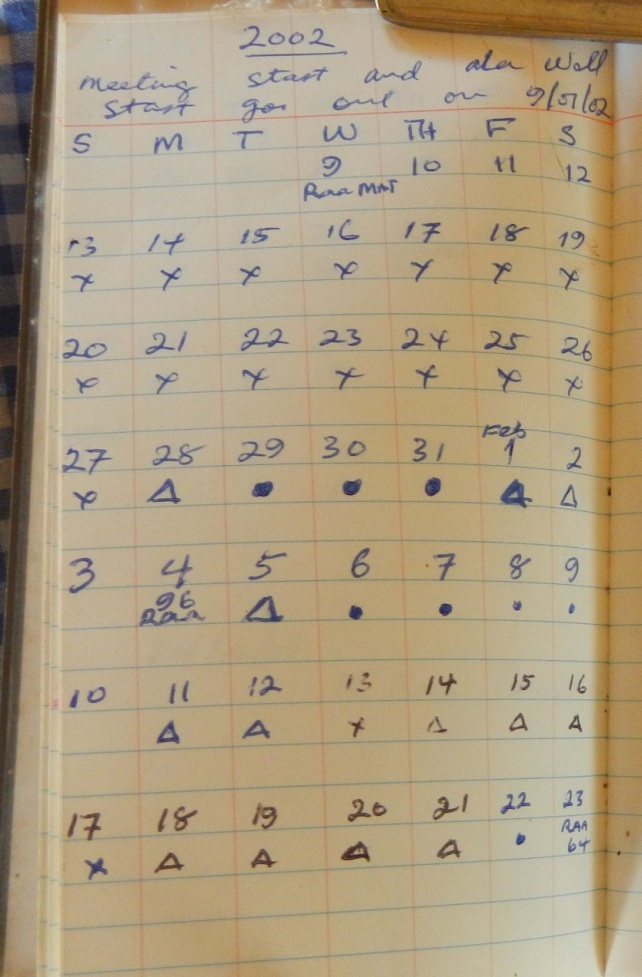

Supplement: Figure S1: Example of catch records from notebook kept by local Fanalei hunter for the period 2000 to early 2003. [file rsos140524supp1.doc]
