# Supplementary material for: Resumption of traditional drive hunting of dolphins in the Solomon Islands in 2013
Source: R Soc Open Sci. 2015 May 6;2(5):140524. doi: 10.1098/rsos.140524 (PMC4453245; doi:10.1098/rsos.140524)
Supplement: Table S1: Catch records from notebook kept by local Fanalei hunter, Albert Balei, for the period 2000 to early 2003. Scientific names of dolphin species follow translations of local names as discussed in the text. [file rsos140524supp2.doc]

Oremus M, Leqata J, Baker CS. 2015. ‘Resumption of traditional drive hunting of dolphins in the Solomon Islands in 2013’, *Proceedings of the Royal Society Open.*

Supplemental Material, Table S1: Catch records from notebook kept by local Fanalei hunter, Albert Balei, for the period 2000 to early 2003. Scientific names of dolphin species follow translations of local names as discussed in the text.

| **Date** | **Go out** | **Sighting** | **Catch** | **Dolphin species** | **Number caught** | **Comment** |
| --- | --- | --- | --- | --- | --- | --- |
| **YEAR 2000** |  |  |  |  |  |  |
| 30/01/00? | Yes | Yes | Yes | *S. longirostris* | 42 | Season Start |
| 1/31/00 | No |  |  |  |  |  |
| 2/1/00 | No |  |  |  |  |  |
| 2/2/00 | No |  |  |  |  |  |
| 2/3/00 | No |  |  |  |  |  |
| 2/4/00 | No |  |  |  |  |  |
| 2/5/00 | No |  |  |  |  |  |
| 2/6/00 | No |  |  |  |  |  |
| 2/7/00 | No |  |  |  |  |  |
| 2/8/00 | No |  |  |  |  |  |
| 2/9/00 | No |  |  |  |  |  |
| 2/10/00 | No |  |  |  |  |  |
| 2/11/00 | No |  |  |  |  |  |
| 2/12/00 | Yes | No |  |  |  |  |
| 2/13/00 | No |  |  |  |  |  |
| 2/14/00 | Yes | No |  |  |  |  |
| 2/15/00 | Yes | Yes | Yes | *S. longirostris* | 15 |  |
| 2/16/00 | Yes | Yes | No |  |  |  |
| 2/17/00 | Yes | Yes | No |  |  |  |
| 2/18/00 | Yes | No |  |  |  |  |
| 2/19/00 | Yes | No |  |  |  |  |
| 2/20/00 | No |  |  |  |  |  |
| 2/21/00 | Yes | No |  |  |  |  |
| 2/22/00 | Yes | Yes | No |  |  |  |
| 2/23/00 | Yes | No |  |  |  |  |
| 2/24/00 | Yes | No |  |  |  |  |
| 2/25/00 | Yes | Yes | Yes | *S. attenuata* | 40 |  |
| 2/26/00 | Yes | No |  |  |  |  |
| 2/27/00 | No |  |  |  |  |  |
| 2/28/00 | Yes | No |  |  |  |  |
| 2/29/00 | Yes | Yes | No |  |  |  |
| 3/1/00 | Yes | No |  |  |  |  |
| 3/2/00 | Yes | Yes | Yes | *S. longirostris* | 55 |  |
| 3/3/00 | Yes | Yes | No |  |  |  |
| 3/4/00 | No |  |  |  |  |  |
| 3/5/00 | No |  |  |  |  |  |
| 3/6/00 | No |  |  |  |  |  |
| 3/7/00 | Yes | Yes | No |  |  |  |
| 3/8/00 | Yes | Yes | Yes | *S. attenuata* | 45 |  |
| 3/9/00 | No |  |  |  |  |  |
| 3/10/00 | Yes | No |  |  |  |  |
| 3/11/00 | Yes | No |  |  |  |  |
| 3/12/00 | No |  |  |  |  |  |
| 3/13/00 | Yes | No |  |  |  |  |
| 3/14/00 | No |  |  |  |  |  |
| 3/15/00 | No |  |  |  |  |  |
| 3/16/00 | No |  |  |  |  |  |
| 3/17/00 | No |  |  |  |  |  |
| 3/18/00 | No |  |  |  |  |  |
| 3/19/00 | No |  |  |  |  |  |
| 3/20/00 | No |  |  |  |  |  |
| 3/21/00 | No |  |  |  |  |  |
| 3/22/00 | No |  |  |  |  |  |
| 3/23/00 | Yes | No |  |  |  |  |
| 3/24/00 | Yes | No |  |  |  |  |
| 3/25/00 | No |  |  |  |  |  |
| 3/26/00 | No |  |  |  |  |  |
| 3/27/00 | Yes | Yes | Yes | *S. longirostris* | 44 |  |
| 3/28/00 | Yes | No |  |  |  |  |
| 3/29/00 | Yes | No |  |  |  |  |
| 3/30/00 | Yes | No |  |  |  |  |
| 3/31/00 | Yes | No |  |  |  |  |
| 4/1/00 | ? |  |  |  |  |  |
| 4/2/00 | No |  |  |  |  |  |
| 4/3/00 | Yes | No |  |  |  |  |
| 4/4/00 | Yes | Yes | No |  |  |  |
| 4/5/00 | Yes | Yes | Yes | *S. attenuata* | 36 |  |
| 4/6/00 | Yes | Yes | Yes | *S. attenuata* | 274 |  |
| 4/7/00 | Yes | No |  |  |  |  |
| 4/8/00 | Yes | No |  |  |  |  |
| 4/9/00 | No |  |  |  |  |  |
| 4/10/00 | No |  |  |  |  |  |
| 4/11/00 | No |  |  |  |  |  |
| 4/12/00 | Yes | No |  |  |  |  |
| 4/13/00 | Yes | No |  |  |  |  |
| 4/14/00 | Yes | No |  |  |  |  |
| 4/15/00 | Yes | Yes | No |  |  |  |
| 4/16/00 | No |  |  |  |  |  |
| 4/17/00 | No |  |  |  |  |  |
| 11/??/00 |  |  |  | *S. longirostris* | 15 | Entered the lagoon alone |
| 12/2/00 |  |  |  | *S. longirostris* | 11 | Found in front of the pass |
| **YEAR 2001** |  |  |  |  |  |  |
| 1/21/01 | No |  |  |  |  | Season start |
| 1/22/01 | No |  |  |  |  |  |
| 1/23/01 | No |  |  |  |  |  |
| 1/24/01 | No |  |  |  |  |  |
| 1/25/01 | No |  |  |  |  |  |
| 1/26/01 | No |  |  |  |  |  |
| 1/27/01 | No |  |  |  |  |  |
| 1/28/01 | No |  |  |  |  |  |
| 1/29/01 | No |  |  |  |  |  |
| 1/30/01 | Yes | No |  |  |  |  |
| 1/31/01 | Yes | Yes | Yes | *S. longirostris* | 19 |  |
| 2/1/01 | No |  |  |  |  |  |
| 2/2/01 | No |  |  |  |  |  |
| 2/3/01 | Yes | Yes | No |  |  |  |
| 2/4/01 | No |  |  |  |  |  |
| 2/5/01 | No |  |  |  |  |  |
| 2/6/01 | Yes | No |  |  |  |  |
| 2/7/01 | No |  |  |  |  |  |
| 2/8/01 | Yes | No |  |  |  |  |
| 2/9/01 | Yes | No |  |  |  |  |
| 2/10/01 | Yes | No |  |  |  |  |
| 2/11/01 | No |  |  |  |  |  |
| 2/12/01 | No |  |  |  |  |  |
| 2/13/01 | Yes | No |  |  |  |  |
| 2/14/01 | Yes | No |  |  |  |  |
| 2/15/01 | Yes | No |  |  |  |  |
| 2/16/01 | Yes | No |  |  |  |  |
| 2/17/01 | Yes | No |  |  |  |  |
| 2/18/01 | No |  |  |  |  |  |
| 2/19/01 | Yes | No |  |  |  |  |
| 2/20/01 | Yes | Yes | Yes | *S. longirostris* | 27 |  |
| 2/21/01 | Yes | No |  |  |  |  |
| 2/22/01 | No |  |  |  |  |  |
| 2/23/01 | No |  |  |  |  |  |
| 2/24/01 | Yes | No |  |  |  |  |
| 2/25/01 | No |  |  |  |  |  |
| 2/26/01 | No |  |  |  |  |  |
| 2/27/01 | No |  |  |  |  |  |
| 2/28/01 | Yes | No |  |  |  |  |
| 3/1/01 | Yes | No |  |  |  |  |
| 3/2/01 | Yes | Yes | No |  |  |  |
| 3/3/01 | Yes | No |  |  |  |  |
| 3/4/01 | No |  |  |  |  |  |
| 3/5/01 | Yes | No |  |  |  |  |
| 3/6/01 | Yes | Yes | No |  |  |  |
| 3/7/01 | Yes | Yes | No |  |  |  |
| 3/8/01 | Yes | No |  |  |  |  |
| 3/9/01 | Yes | No |  |  |  |  |
| 3/10/01 | Yes | No |  |  |  |  |
| 3/11/01 | No |  |  |  |  |  |
| 3/12/01 | Yes | Yes | No |  |  |  |
| 3/13/01 | Yes | Yes | No |  |  |  |
| 3/14/01 | Yes | Yes | No |  |  |  |
| 3/15/01 | Yes | No |  |  |  |  |
| 3/16/01 | No |  |  |  |  |  |
| 3/17/01 | Yes | Yes | Yes | *S. longirostris* | 54 |  |
| 3/18/01 | No |  |  |  |  |  |
| 3/19/01 | Yes | No |  |  |  |  |
| 3/20/01 | Yes | No |  |  |  |  |
| 3/21/01 | Yes | Yes | No |  |  |  |
| 3/22/01 | Yes | Yes | Yes | *S. attenuata* | 25 |  |
| 3/23/01 | Yes | No |  |  |  |  |
| 3/24/01 | Yes | No |  |  |  |  |
| 3/25/01 | No |  |  |  |  |  |
| 3/26/01 | Yes | No |  |  |  |  |
| 3/27/01 | Yes | No |  |  |  |  |
| 3/28/01 | Yes | No |  |  |  |  |
| 3/29/01 | Yes | No |  |  |  |  |
| 3/30/01 | Yes | No |  |  |  |  |
| 3/31/01 | Yes | No |  |  |  |  |
| 4/1/01 | No |  |  |  |  |  |
| 4/2/01 | Yes | No |  |  |  |  |
| 4/3/01 | Yes | No |  |  |  |  |
| 4/4/01 | Yes | Yes | No |  |  |  |
| 4/5/01 | Yes | No |  |  |  |  |
| 4/6/01 | Yes | No |  |  |  |  |
| 4/7/01 | Yes | No |  |  |  | Season End |
| 12/24/07 |  |  |  | *S. longirostris* | 16 | Entered the lagoon alone |
| **YEAR 2002** |  |  |  |  |  |  |
| 1/9/02 | Yes | Yes | Yes | *S. longirostris* | 9 | Season Start |
| 1/10/02 | ? |  |  |  |  |  |
| 1/11/02 | ? |  |  |  |  |  |
| 1/12/02 | ? |  |  |  |  |  |
| 1/13/02 | No |  |  |  |  |  |
| 1/14/02 | No |  |  |  |  |  |
| 1/15/02 | No |  |  |  |  |  |
| 1/16/02 | No |  |  |  |  |  |
| 1/17/02 | No |  |  |  |  |  |
| 1/18/02 | No |  |  |  |  |  |
| 1/19/02 | No |  |  |  |  |  |
| 1/20/02 | No |  |  |  |  |  |
| 1/21/02 | No |  |  |  |  |  |
| 1/22/02 | No |  |  |  |  |  |
| 1/23/02 | No |  |  |  |  |  |
| 1/24/02 | No |  |  |  |  |  |
| 1/25/02 | No |  |  |  |  |  |
| 1/26/02 | No |  |  |  |  |  |
| 1/27/02 | No |  |  |  |  |  |
| 1/28/02 | Yes | Yes | No |  |  |  |
| 1/29/02 | Yes | No |  |  |  |  |
| 1/30/02 | Yes | No |  |  |  |  |
| 1/31/02 | Yes | No |  |  |  |  |
| 2/1/02 | Yes | Yes | No |  |  |  |
| 2/2/02 | Yes | Yes | No |  |  |  |
| 2/3/02 | No |  |  |  |  |  |
| 2/4/02 | Yes | Yes | Yes | *S. longirostris* | 96 |  |
| 2/5/02 | Yes | Yes | No |  |  |  |
| 2/6/02 | Yes | No |  |  |  |  |
| 2/7/02 | Yes | No |  |  |  |  |
| 2/8/02 | Yes | No |  |  |  |  |
| 2/9/02 | Yes | No |  |  |  |  |
| 2/10/02 | No |  |  |  |  |  |
| 2/11/02 | Yes | Yes | No |  |  |  |
| 2/12/02 | Yes | Yes | No |  |  |  |
| 2/13/02 | No |  |  |  |  |  |
| 2/14/02 | Yes | Yes | No |  |  |  |
| 2/15/02 | Yes | Yes | No |  |  |  |
| 2/16/02 | Yes | Yes | No |  |  |  |
| 2/17/02 | No |  |  |  |  |  |
| 2/18/02 | Yes | Yes | No |  |  |  |
| 2/19/02 | Yes | Yes | No |  |  |  |
| 2/20/02 | Yes | Yes | No |  |  |  |
| 2/21/02 | Yes | Yes | No |  |  |  |
| 2/22/02 | Yes | No |  |  |  |  |
| 2/23/02 | Yes | Yes | Yes | *S. longirostris* | 64 |  |
| 2/24/02 | No |  |  |  |  |  |
| 2/25/02 | Yes | No |  |  |  |  |
| 2/26/02 | No |  |  |  |  |  |
| 2/27/02 | Yes | Yes | No |  |  |  |
| 2/28/02 | Yes | Yes | No |  |  |  |
| 3/1/02 | Yes | No |  |  |  |  |
| 3/2/02 | Yes | No |  |  |  |  |
| 3/3/02 | No |  |  |  |  |  |
| 3/4/02 | Yes | No |  |  |  |  |
| 3/5/02 | Yes | No |  |  |  |  |
| 3/6/02 | Yes | Yes | Yes | *S. attenuata* | 18 |  |
| 3/7/02 | Yes | No |  |  |  |  |
| 3/8/02 | Yes | Yes | Yes | *S. longirostris* | 128 |  |
| 3/9/02 | Yes | No |  |  |  |  |
| 3/10/02 | No |  |  |  |  |  |
| 3/11/02 | Yes | No |  |  |  |  |
| 3/12/02 | Yes | No |  |  |  |  |
| 3/13/02 | Yes | Yes | No |  |  |  |
| 3/14/02 | Yes | No |  |  |  |  |
| 3/15/02 | Yes | Yes | No |  |  |  |
| 3/16/02 | Yes | No |  |  |  |  |
| 3/17/02 | No |  |  |  |  |  |
| 3/18/02 | Yes | Yes | Yes | *S. attenuata* | 50 |  |
| 3/19/02 | No |  |  |  |  |  |
| 3/20/02 | No |  |  |  |  |  |
| 3/21/02 | No |  |  |  |  |  |
| 3/22/02 | No |  |  |  |  |  |
| 3/23/02 | Yes | No |  |  |  |  |
| 3/24/02 | No |  |  |  |  |  |
| 3/25/02 | Yes | Yes | Yes | *S. attenuata* | 13 |  |
| 3/26/02 | Yes | No |  |  |  |  |
| 3/27/02 | Yes | Yes | No |  |  |  |
| 3/28/02 | Yes | No |  |  |  |  |
| 3/29/02 | No |  |  |  |  |  |
| 3/30/02 | Yes | Yes | Yes | *S. longirostris* | 33 |  |
| 3/31/02 | No |  |  |  |  |  |
| 4/1/02 | Yes | No |  |  |  |  |
| 4/2/02 | No |  |  |  |  |  |
| 4/3/02 | Yes | No |  |  |  |  |
| 4/4/02 | Yes | No |  |  |  |  |
| 4/5/02 | Yes | No |  |  |  |  |
| 4/6/02 | Yes | No |  |  |  |  |
| 4/7/02 | No |  |  |  |  |  |
| 4/8/02 | Yes | Yes | Yes | *S. attenuata* | 72 |  |
| 4/9/02 | Yes | Yes | Yes | *S. attenuata* | 40 |  |
| 4/10/02 | Yes | No |  |  |  |  |
| 4/11/02 | Yes | No |  |  |  |  |
| 4/12/02 | Yes | No |  |  |  |  |
| 4/13/02 | Yes | No |  |  |  |  |
| 4/14/02 | No |  |  |  |  |  |
| 4/15/02 | Yes | Yes | No |  |  |  |
| 4/16/02 | Yes | No |  |  |  |  |
| 4/17/02 | Yes | Yes | Yes | *S. attenuata* | 125 |  |
| 4/18/02 | No |  |  |  |  |  |
| 4/19/02 | ? |  |  |  |  |  |
| 4/20/02 | Yes | Yes | No |  |  |  |
| 4/21/02 | No |  |  |  |  |  |
| 4/22/02 | Yes | Yes | No |  |  |  |
| 4/23/02 | Yes | No |  |  |  |  |
| 4/24/02 | Yes | Yes | No |  |  |  |
| 4/25/02 | Yes | No |  |  |  |  |
| 4/26/02 | Yes | Yes | No |  |  |  |
| 4/27/02 | No |  |  |  |  |  |
| **YEAR 2003** |  |  |  |  |  |  |
| 2/26/03 | Yes | No |  |  |  |  |
| 2/27/03 | Yes | No |  |  |  |  |
| 2/28/03 | Yes | No |  |  |  |  |
| 3/1/03 | Yes | No |  |  |  |  |
| 3/2/03 | No |  |  |  |  |  |
| 3/3/03 | Yes | Yes | No |  |  |  |
| 3/4/03 | Yes | Yes | No |  |  |  |
| 3/5/03 | Yes | No |  |  |  |  |
| 3/6/03 | Yes | No |  |  |  |  |
| 3/7/03 | Yes | No |  |  |  |  |
| 3/8/03 | Yes | No |  |  |  |  |
| 3/9/03 | No |  |  |  |  |  |
| 3/10/03 | No |  |  |  |  |  |
| 3/11/03 | No |  |  |  |  |  |
| 3/12/03 | No |  |  |  |  |  |
| 3/13/03 | No |  |  |  |  |  |
| 3/14/03 | No |  |  |  |  |  |
| 3/15/03 | No |  |  |  |  |  |
| 3/16/03 | No |  |  |  |  |  |
| 3/17/03 | Yes | Yes | No |  |  |  |
| 3/18/03 | Yes | No |  |  |  |  |
| 3/19/03 | Yes | Yes | No |  |  |  |
| 3/20/03 | Yes | Yes | No |  |  |  |
| 3/21/03 | Yes | No |  |  |  |  |
| 3/22/03 | No |  |  |  |  |  |
| 3/23/03 | No |  |  |  |  |  |
| 3/24/03 | No |  |  |  |  |  |
| 3/25/03 | Yes | No |  |  |  |  |
| 3/26/03 | Yes | No |  |  |  |  |
| 3/27/03 | ? |  |  |  |  |  |
| 3/28/03 | ? |  |  |  |  |  |
| 3/29/03 | ? |  |  |  |  |  |
| 3/30/03 | No |  |  |  |  |  |
| 3/31/03 | Yes | No |  |  |  |  |
| 4/1/03 | Yes | No |  |  |  |  |
| 4/2/03 | Yes | No |  |  |  |  |
| 4/3/03 | Yes | No |  |  |  |  |
| 4/4/03 | No |  |  |  |  |  |
| 4/5/03 | No |  |  |  |  |  |
| 4/6/03 | No |  |  |  |  |  |
| 4/7/03 | Yes | No |  |  |  |  |
| 4/8/03 | Yes | No |  |  |  |  |
| 4/9/03 | Yes | No |  |  |  |  |
| 4/10/03 | Yes | No |  |  |  |  |
| 4/11/03 | Yes | No |  |  |  |  |
| 4/12/03 | Yes | No |  |  |  |  |
| 4/13/03 | No |  |  |  |  |  |
| 4/14/03 | Yes | Yes | Yes | *S. attenuata* | 400 |  |
| 4/15/03 | No |  |  |  |  |  |
| 4/16/03 | No |  |  |  |  |  |
| 4/17/03 | Yes | No |  |  |  |  |
| 4/18/03 | Yes | No |  |  |  |  |
| 4/19/03 | Yes | No |  |  |  |  |
|  |  |  |  |  |  |  |
